# Converging Strategies in Expression of Human Complex Retroviruses

**DOI:** 10.3390/v3081395

**Published:** 2011-08-11

**Authors:** Ilaria Cavallari, Francesca Rende, Donna M. D'Agostino, Vincenzo Ciminale

**Affiliations:** 1 Department of Oncology and Surgical Sciences, University of Padova, Via Gattamelata 64, I-35128 Padova, Italy; E-Mails: ilaria.cavallari@unipd.it (I.C.); francesca.rende@unipd.it (F.R.); dm.dagostino@unipd.it (D.M.D.); 2 Istituto Oncologico Veneto-IRCCS, I-35128 Padova, Italy

**Keywords:** HTLV-1, Rex, leukemia

## Abstract

The discovery of human retroviruses in the early 1980s revealed the existence of viral-encoded non-structural genes that were not evident in previously described animal retroviruses. Based on the absence or presence of these additional genes retroviruses were classified as ‘simple’ and ‘complex’, respectively. Expression of most of these extra genes is achieved through the generation of alternatively spliced mRNAs. The present review summarizes the genetic organization and expression strategies of human complex retroviruses and highlights the converging mechanisms controlling their life cycles.

## 1. Introduction

Retroviruses are distinguished by a replication cycle that relies on reverse transcription of an RNA genome and integration of the resulting double-stranded DNA provirus into the genome of the host; expression of the integrated provirus gives rise to new RNA genomes and mRNAs that are translated into structural proteins and enzymes necessary for assembly of new virions. All retroviruses contain the gag, pro, pol and env genes coding for virion structural proteins and enzymes necessary for protein processing, reverse transcription and integration. All these genes are coded on the plus-strand of the proviral genome. The Gag, Pro and Pol open reading frames (ORFs), coded in the unspliced primary transcript, are partially overlapping and rely on one or two ribosomal frameshifting events for translation, while Env is produced from a separate singly spliced mRNA. Other conserved elements include long terminal repeats (LTRs) which direct transcription, polyadenylation and integration, a packaging signal that permits incorporation of the RNA genome into virions, and a primer binding site and polypurine tract that are needed for synthesis of the minus and plus strands of the provirus [1].

The discovery of the human T-cell leukemia viruses (HTLVs) and human immunodeficiency viruses (HIVs) in the early 1980s revealed the existence of additional viral-encoded non-structural genes and regulatory elements that were not evident in previously described animal retroviruses. Based on the absence or presence of these additional genes retroviruses were classified as ‘simple’ and ‘complex’, respectively [2]. Complex retroviruses comprise the Lentivirus, Deltaretrovirus, Epsilonretrovirus, and Spumavirus genera. Although Betaretroviruses were long considered to be simple, the presence of extra genes and virus-directed post-transcriptional regulatory mechanisms in the Betaretroviruses mouse murine leukemia virus (MMTV) [3,4] and HERV-K family of human endogenous retroviruses [5] indicates a need to update this classification.

For the most part, the extra genes of complex retroviruses are coded on the plus-strand of the provirus, with transcription from the 5′LTR promoter producing a primary transcript that undergoes alternative splicing. Further layers of complexity can be found in foamy viruses, which possess an alternative plus-strand promoter, and in HTLV and HIV, which produce minus-strand transcripts from promoters in the 3′LTR. Among the extra gene products that define complex retroviruses, HIV Tat and Rev and their orthologs in other complex retroviruses stand out as key regulatory proteins that orchestrate viral gene expression.

Common to the expression strategies of all retroviruses is the necessity to export and translate a proportion of incompletely spliced mRNAs; this requires both a relative inefficiency of viral mRNA splicing and the evasion of cellular mechanisms mediating nuclear retention and degradation of intron-containing transcripts. For some simple retroviruses such as Mason-Pfizer Monkey virus (MPMV), this is accomplished through the interaction of a *cis*-acting RNA element termed the constitutive transport element (CTE) with the cellular nuclear export factors NXF1/NXT1 [6]. As described in greater detail below, most of the complex retroviruses code for both a *cis*-acting regulatory element(s) and post-transcriptional regulatory proteins, whose binding to distinct stem-loop elements present in the viral mRNAs forms a bridge that allows interaction of the RNA with cellular factors mediating nucleo-cytoplasmic export.

The patterns of alternatively spliced, plus and minus-strand mRNAs and production of essential regulatory proteins suggest a temporal regulation of viral expression and the possibility that different expression patterns may be associated with different infection states and disease outcomes. Such temporal regulation of viral expression has been thoroughly characterized in DNA tumor viruses such as herpesviruses, where different patterns of viral gene expression are associated with latent or productive phases of the viral life cycle and with different diseases (reviewed in [7]). In the case of Epstein-Barr virus (EBV), the switch between early and late viral genes is achieved mainly through the genetic and epigenetic regulation of alternative viral promoters. Human papillomaviruses (HPV) exploit a combination of alternative promoter usage, alternative splicing and selection of “early” *vs.* “late” polyadenylation sites (reviewed in [8]). The early/late pattern of HPV expression is associated with tissue tropism and restriction of the lytic cycle to differentiated epithelia (reviewed in [9]).

In the present review, the lentivirus HIV-1 and the deltaretrovirus HTLV-1 are used as points of reference to compare the expression strategies of complex retroviruses in terms of transcript profiles, roles of regulatory proteins and temporal control of transcript expression.

## 2. Expression Strategies of HIV-1

Early studies of HIV-1, the causative agent of acquired immunodeficiency syndrome (AIDS), revealed 3 classes of viral transcripts visible by northern blotting: (i) “full length” unspliced mRNA of about 9 kb; (ii) “intermediate” mRNAs of about 4 kb that included the singly-spliced env transcript; (iii) and “small” mRNAs of about 2 kb comprising several multiply spliced species [10]. Subsequent RT-PCR-based methods aimed at identifying transcripts coding for the virus’s 6 extra proteins (Tat, Rev, Nef, Vif, Vpr, and Vpu) revealed the production of over 40 alternatively-spliced plus-strand mRNAs with partially overlapping coding potentials [11,12]. The 4 kb class of viral mRNAs consists of at least 12 differentially spliced species—nine are bicistronic mRNAs producing Env and Vpu and three mRNAs express a 1-exon isoform of Tat [13,14]. The fact that Env and Vpu proteins are expressed from the same Rev-dependent mRNAs suggests a coordinate expression of these proteins; a distinct set of intermediate size, Rev-dependent mRNAs encode Vif and Vpr [13,14]. Purcell and Martin described an even more complex pattern of alternatively spliced mRNAs, with 12 mRNAs encoding Rev, 5 transcripts encoding Nef, 8 encoding Tat and 16 encoding Env [11]. In addition, transcription of the minus strand of HIV gives rise to a transcript that was detected in chronically-infected cell lines and in PBMCs of HIV-infected patients; this mRNA contains an ORF with the potential to code for a hydrophobic protein termed ASP [15].

Optimal expression of the alternatively spliced HIV mRNAs results from the concerted action of *cis*-acting RNA elements and *trans*-acting viral/cellular proteins. The former include several exonic splicing enhancers (ESE) and silencers (ESS) which play a crucial role in determining the “strength” and probability of selection of 5′splice site (SS) and 3′SS pairs resulting in exon definition. The relative abundance of the different HIV mRNAs is strictly dependent on their consensus sequence and on their regulation by ESE and ESS which exert their functions by binding cellular proteins of the SR and hRNP-A/B families [16,17].

### Positive regulators of viral gene expression

Balanced expression of the 3 size classes of mRNAs is achieved through the concerted action of the two essential viral regulatory proteins, Tat and Rev. Tat drives transcription from the viral LTR promoter by binding to a stem-loop RNA sequence present at the 5′ end of all viral transcripts. This interaction results in hyperphosphorylation of the C-terminal tail of RNA polymerase II resulting in enhanced transcriptional elongation; through this mechanism Tat provides a positive-feedback loop controlling overall HIV expression [18]. The minus-strand promoter is also responsive to Tat transactivation, although to a lesser extent than the plus-strand promoter [19].

Recent evidence suggests that Tat also influences HIV expression at the level of splicing. In particular, Berro *et al.* showed that acetylated Tat binds to the p32 cellular protein, resulting in inhibition the activating phosphorylation of the SF2 splicing factor by the CDK13 kinase [20,21]. Through this function, Tat would thus play a role in HIV expression by maintaining viral splicing efficiency at a low level, a key feature that is required for the function of Rev [22].

**Figure 1.**
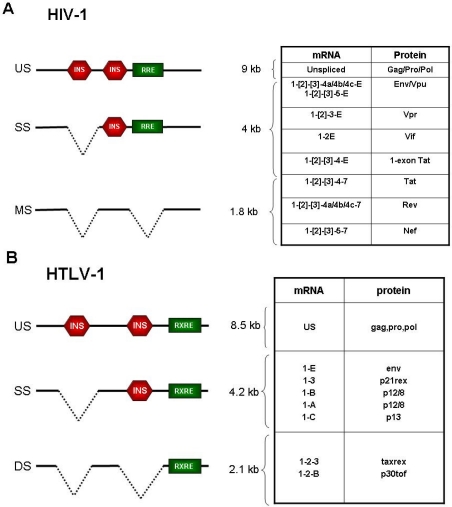
Exon composition and coding potential of the main plus-strand mRNAs of HIV-1 (reviewed in [16]) (**A**) and HTLV-1 (**B**). The INhibitory Sequences (INS) and the Rev/Rex-Responsive Element (RRE/RXRE) are indicated by red hexagons and green boxes, respectively.

Rev interacts with an RNA element termed the Rev-responsive element (RRE) located in the env region of the genome. Engagement of viral transcripts by Rev permits their exit from the nucleus by the export factor CRM1, thus subtracting them from the splicing machinery, which would otherwise result in removal of the RRE (Figure 1A). Rev-dependent mRNAs include the unspliced transcript which serves as the RNA genome and codes for the Gag-Pro-Pol proteins, and the series of singly spliced mRNAs coding for Vif, Vpr, Vpu, and Env proteins (reviewed in [23]). The RRE is spliced out of the multiply-spliced Rev-independent transcripts; these mRNAs include those producing Tat, Nef and Rev itself. In addition to affecting RNA export, Rev promotes loading of the responsive mRNAs onto polysomes and enhances encapsidation of the genomic RNA [24].

### Inhibitory elements

*Cis*-acting sequences that negatively affect transcript expression have been mapped to the HIV-1 gag, pol and env genes (Figure 1A) [25–27]. Maldarelli *et al.* showed that 2 inhibitory sequences present in the gag and pol regions mediate nuclear retention of the mRNAs [26]. Schwartz *et al.* mapped distinct elements in the gag-protease genes that act by decreasing RNA stability [28]; this inhibitory effect is counteracted by the Rev-RRE interaction, which promotes nuclear export of the mRNAs [29]. These sequences, which, like the RRE, are absent in the multiply spliced, Rev-independent mRNAs, do not contain splice sites nor appear to act on splicing, but are rich in AU nucleotides, a feature that is common to cellular transcripts with short half-lives. Mutation of these AU-rich sequences results in Rev-independent gag expression [30]. Using laser-scanning confocal microscopy, Berthold and Maldarellli showed that, in the absence of Rev, *cis*-acting inhibitory elements present in the gag gene direct viral transcripts to small nuclear granules, while viral transcripts lacking these sequences (*i.e.*, multiply spliced mRNAs) are localized in distinct large nuclear clusters containing the SC35 splicing factor [31]. It is not known whether these properties are shared by the *cis*-acting inhibitory sequences identified in the env gene, although their inhibitory function is not attributable to the presence of splice sites [25,27].

### Kinetics of gene expression

Early studies suggested that Rev function provides a molecular switch between latent and productive infection. Using a single-cycle infection model in the CD4+ T-cell line H9, Kim *et al.* showed a Rev-dependent temporal pattern in the expression of the different classes of HIV transcripts, with the 2-kilobase multiply-spliced mRNA group (encoding Tat, Rev, Nef) expressed earliest. The 9-kb and, to a lesser extent, the 4-kb classes of transcripts were detected with a delay of about 12 hours [32]. Consistent with these findings, Ahmad *et al.* showed that a Rev-mutant virus was replication-deficient and exhibited an expression pattern characterized by the accumulation of Nef [33]. Taken together, these studies strongly suggest that the HIV-1 life cycle is characterized by a two-phase kinetics with Tat, Rev, and Nef expressed as early genes and Env, Vpu, Vif, Vpr, Gag, Pro, and Pol expressed as late genes.

In addition to helping dissect the mechanisms of viral gene expression, these studies provided clues to the mechanisms controlling viral latency, a key strategy employed by many viruses to establish life-long persistence in the host. Although viral expression is not completely silenced during the early phase, it can be considered a form of viral latency, since the proteins and genomic RNA forming infectious virions cannot be produced. Prolonged viral latency may result from a blockade of this switch, *i.e.*, through an inhibition of Rev function. More recent studies indicate that modulation of Tat activity (e.g., through acetylation) may also play a key role in determining HIV latency, resulting in a substantial decrease in overall viral expression [34–36].

The timing and relative expression of the different viral genes in relation to the host cell and clinical status of the patient is of particular relevance in light of the diverse functions of the regulatory and accessory genes, which play an essential role in controlling viral expression and in counteracting antiviral host restriction factors and circumventing innate and adaptive immune responses, thus favouring viral persistence and spread [37,38].

Nef, which is expressed as an early Rev-independent gene, plays an important role in HIV pathogenesis, as demonstrated by the long-term survival of patients infected with Nef-defective HIV strains [39]. Nef enhances the clathrin-mediated endocytosis and degradation of CD4, the primary receptor for HIV entry [40]. Vpu, whose expression is Rev-dependent and tied to that of Env, also interacts with CD4, resulting in its retention in the endoplasmic reticulum (ER), polyubiquitylation and proteasomal degradation. The fact that two viral proteins—Nef and Vpu—target CD4 for degradation suggests the importance of CD4 downregulation for viral propagation, possibly at the levels of nascent virus assembly and release [41,42]. Vpu also promotes release of nascent virions by inhibiting tetherin, a surface glycoprotein that forms connections between the plasma membrane and virion envelope which hamper release of viral particles from infected cells [43].

Nef also sabotages the host’s antiviral defenses by downregulating surface expression of MHC class I and T cell receptor (TCR)-CD3 complexes, the former resulting in impaired recognition of infected cells by CTLs (cytotoxic T-lymphocytes) and the latter reducing the efficiency of “immunological synapse” formation between infected cells and antigen-presenting cells [44]. The Vif protein also plays a key role in curbing antiviral host defenses by directing proteasomal degradation of APOBEC3G (apolipoprotein B mRNA-editing enzyme catalytic polypeptide-like 3G), an enzyme that induces hypermutation of the viral genome by deaminating C residues in the nascent minus-strand product of reverse transcription [45]. Vpr exerts cytopathic effects mediated by its ability to affect mitochondrial function, increases viral transcription and induces cell-cycle arrest in the G2 phase [37]. Vpr is also incorporated into viral particles and facilitates transport of the preintegration complex into the nucleus, a function that was proposed to be particularly relevant in infection and persistence in non-dividing cells such as macrophages [46] (Table 1).

In analogy to HIV, animal lentiviruses such as OMVV (ovine maedi-visna virus), CAEV (caprine arthritis encephalitis virus) and EIAV (equine infectious anemia virus) also express several multiply spliced mRNAs encoding regulatory proteins, with balanced transcript expression controlled by Rev orthologs and cognate Rev-responsive elements (RRE). Schoborg *et al.* [47] demonstrated a “two-phase” temporal pattern of viral gene expression in CAEV-infected cells, with Rev expressed as an early gene and Gag expressed as a late gene. These data are consistent with findings from studies of OMVV-infected sheep cells, where expression of small multiply-spliced transcripts (encoding Rev and Tat orthologs) precedes that of transcripts encoding Gag and Env [48]. In the case of EIAV, although the function of EIAV-Rev is analogous to that of HIV-1 Rev, there are significant differences in the *cis*- and *trans*-acting components [49]. Martarano *et al.* [49] demonstrated that, in the absence of EIAV Rev, the mRNA containing exons 1-2-3-4, encoding both Tat and Rev, is predominant, while in the presence of Rev, exon 3 is skipped and the resulting mRNA 1-2-4 produces Tat.

## 3. Expression Strategies of HTLV-1

HTLV-1 is the causative agent of adult T-cell leukemia/lymphoma (ATLL), an aggressive malignancy of mature CD4+ T-cells, and of tropical spastic paraparesis/HTLV-1-associated myelopathy (TSP/HAM), a demyelinating neurodegenerative disease. HTLV-1 exhibits a complex genetic organization and expression strategy characterized by the production of several additional regulatory and accessory genes located at the 3′ end of the genome (the pX region), characteristics that are shared with the other deltaretroviruses (HTLV-2, STLVs and BLV). The pX region of HTLV-1 contains four major overlapping ORFs, termed x-I through x-IV, coding for the regulatory proteins Tax and Rex and the accessory proteins p12, p13, p30/Tof and p21Rex [50,51]. The minus strand of HTLV-1 also contains an ORF located in the pX region [52] which codes for the HBZ protein (HTLV-1 bZIP factor) [53].

As in HIV, expression of the highly condensed genetic information of HTLV-1 is achieved through ribosomal frameshifting (which generates a Gag-Pro-Pol polyprotein from the full-length transcript), polycistronic translation (which directs production of Tax and Rex from the same mRNA) and alternative splicing. Minus-strand transcription generates at least 2 alternatively spliced transcripts coding for 2 isoforms of the HBZ protein [52,53]. The HTLV-1 transcripts can be grouped in 4 major classes: (a) genomic unspliced mRNA, coding for Gag-Pro-Pol; (b) singly-spliced mRNAs, coding for the Envelope glycoproteins and for the accessory proteins p21Rex, p12 and p13; (c) doubly-spliced mRNAs, coding for the regulatory proteins p40 (Tax) and p27 (Rex), and for the regulatory/accessory protein p30Tof; (d) mRNAs generated from minus strand transcription, coding for HBZ.

### Positive regulators of viral gene expression

HTLV-1 expression is controlled by two regulatory circuits: a positive feedback loop provided by the viral transactivator Tax that drives transcription of the viral genome [54], and a post-transcriptional regulatory loop provided by Rex which, by binding to the Rex-responsive element (RXRE) present at the 3′ end of all plus-strand HTLV-1 transcripts, enhances the nuclear export and expression of a subset of mRNAs [55,56]. In analogy to HIV-1 Rev, the effect of Rex on the nucleo-cytoplasmic export of viral mRNAs is mediated through binding to CRM1 [57]. Interestingly, while HTLV-1 Rex can also function on the RRE of HIV-1, HIV-1 Rev is unable to act on the HTLV-I RXRE [58].

### Inhibitory elements

As in the case of HIV, negative *cis*-acting inhibitory elements (INS) within the gag-pol and env regions of HTLV-1 (Figure 1B) are at least in part responsible for the Rex dependence of the full-length mRNA and the singly spliced env mRNAs [59]. This inhibitory effect is not affected by Tax, but is relieved by binding of Rex to the RXRE, suggesting that the INS act at the RNA level.

### Kinetics of gene expression

The study of the kinetics of viral expression in HTLV-1 is difficult due to its relative inefficiency in infecting target cells in “cell-free” *in vitro* systems. This limitation has prevented the use of single-cycle infection systems employed for HIV-1. The first study aimed at investigating the kinetics and regulation of HTLV-1 gene expression was based on the transient transfection of an HTLV-1 molecular clone followed by Northern blotting of the polyadenylated, cytoplasmic mRNA fraction [55]. Results revealed three bands of 8.5, 4.2 and 2.1 kb, corresponding to unspliced, singly-spliced and doubly-spliced transcripts, respectively. Interestingly, only the 2.1-kb mRNA was detected early after transfection (10 hours), while the 8.5-kb and 4.2-kb mRNAs were detected at 16 hours. At later time points expression of the 8.5-kb and 4.2-kb bands increased while the 2.1-kb band decreased slightly. Fifty-two hours after transfection the 2.1-kb mRNA was barely detectable, while the 8.5-kb and 4.1-kb mRNAs were still detected. Based on these findings, the investigators indicated three stages of HTLV-1 expression: “early”, in which only the 2.1-kb mRNA is present; “intermediate”, in which all three classes of viral mRNAs are present; “late” in which only the 8.5-kb and 4.2-kb transcripts are detected. In cells transfected with a Rex-mutant molecular clone, only the 2.1-kb mRNA was detectable 40 hours after transfection, demonstrating that Rex is essential for accumulation of the 8.5-kb and 4.2-kb mRNAs.

Although these early studies showed a qualitative switch among classes of HTLV-1 mRNAs (multiply-spliced *vs.* unspliced/singly-spliced mRNAs), the detection of a temporal switch in the expression of individual viral transcripts proved difficult to demonstrate experimentally using quantitative transcript-specific Real Time RT-PCR [60,61]. A study performed using PBMCs obtained from HTLV-1-inoculated rabbits indicated early expression of gag/pol, tax/rex and env mRNAs, whereas the accessory genes and the antisense HBZ mRNAs were expressed late [61]. Such early expression of gag/pol and env mRNAs was unexpected considering earlier results based on Northern blotting [55]. Analysis of the kinetics of HTLV-1 gene expression in the rabbits indicated that the tax/rex and gag/pol mRNAs were expressed very early after inoculation and then progressively decreased, while the HBZ mRNA was present at low levels at early time points and slowly increased and stabilized over time in direct correlation with the proviral load [61].

We recently investigated the temporal sequence of HTLV-1 gene expression using splice site-specific Real Time RT-PCR in an *ex vivo* virus reactivation model based on the depletion of CD8+ T cells from unstimulated PBMCs isolated from HTLV-1-infected patients (ATLL and TSP/HAM) [62]. The results indicated a “two-phase” kinetics with tax/rex expression preceding that of all other viral transcripts, suggesting an “early-late” switch in HTLV-1 gene expression. Analysis of cells transfected with HTLV-1 molecular clones demonstrated the strict Rex-dependency of this “two-phase” kinetics. Mathematical modeling indicated that the observed two-phase kinetics must be critically dependent on a delay of Rex function compared to Tax [63]. This prediction was supported by experimental evidence demonstrating a delayed accumulation and a longer half-life of Rex compared to Tax [62].

The expansion and survival of HTLV-1-infected T-cells can be attributed primarily to Tax, whose activity as a modulator of a variety of transcription factors and associations with components of signal transduction pathways alter expression of host-cell genes involved in proliferation, genetic stability, and apoptosis (reviewed by [64–66]). The other X region genes also exhibit interesting (and in some cases opposite) effects on viral replication, infected-cell turnover and persistence in the host (Table 2). Changes in the relative abundance and timing of expression of these genes may therefore have important effects on the outcome of infection.

p30Tof is a nucleolar/nuclear non-shuttling protein [67] that inhibits the nuclear export of the tax/rex mRNA; this effect results in a global inhibition of viral gene expression, suggesting that it might act as a latency factor [68,69]. p30Tof also affects Tax-mediated transcription by interacting with the co-activator CBP/p300 [70,71] and influences the expression of a number of cellular genes at the transcriptional and post-transcriptional levels, including genes involved in T-cell activation and apoptosis [72,73]. In addition, p30Tof was found to interact with the RNA-binding domain of Rex and prevent Rex interacting with the RxRE [74]. p30Tof also interacts with PU.1 and inhibits its transcriptional activity, resulting in the down-regulation of Toll-like receptor 4 (TLR4) expression from the cell surface [75].

Another HTLV-1 protein that is proposed to act as a latency-inducing factor is p21Rex, a truncated isoform of Rex lacking the N-terminal RNA-binding and multimerization domains of the full-length protein. p21Rex was proposed to act as a repressor of full-length Rex [76,77], resulting in a reduction in the expression of the transcripts coding for the structural viral proteins.

p13, an 87-amino acid protein that corresponds to the C-terminal portion of p30Tof [51], accumulates in the mitochondrial inner membrane [78] and induces alterations in mitochondrial function [78,79], cell turnover [80,81] and tumor growth in *in vivo* experimental models [80]. Recent studies suggest that by controlling mitochondrial ROS production, p13 may have a distinct impact on cell survival and proliferation depending on the cell’s inherent ROS set-point, with activation predominating in normal resting T-cells and death-promoting effects in transformed cells [82,83]. In the presence of Tax, p13 is stabilized and partially targeted to the nucleus, where it binds Tax and inhibits its transcriptional activity [83,84].

p12 accumulates in the ER and Golgi apparatus, where it interacts with the β and γ_c_ chains of the interleukin-2 receptor (IL-2R), resulting in reduced surface expression [85] and an increase in the transcriptional activity of STAT-5, thereby providing a proliferative advantage to T cells [86]. p12 also decreases surface expression of MHC-I, thus contributing to blunt CTL recognition of HTLV-1 infected cells [87]. These effects on the surface expression of key T-cell signaling molecules are reminiscent of HIV Nef (see above). p12 also interacts with calreticulin and calnexin [88], resulting in Ca^2+^ leakage from the ER [89] and NFAT activation [90,91]; taken together, these effects decrease the activation threshold of HTLV-1-infected T-cells [92]. Proteolytic cleavage of p12 in the ER yields an 8-kDa protein named p8 which traffics to the cell surface and is recruited to the immunological synapse, resulting in promotion of T-cell anergy; it also triggers the formation of conduits among neighboring T-cells, resulting in increased virus transmission [93,94]. This latter function of p8 is reminiscent of “long-range intercellular nanotubes” induced by HIV Nef [95]. Although HIV-1 and HTLV-1 have clearly distinct pathogenic properties, the functional analogies among their regulatory and accessory proteins highlight common strategies that these viruses must have evolved for life-long persistence in the host.

The HBZ protein interacts with a number of transcription factors, including CREB-2, p300/CBP, Jun family members, and NF-κB [96]. HBZ inhibits Tax-mediated viral transcription [53,97] and promotes proliferation of ATLL cells *in vivo* and *in vitro* [98,99]. The hbz mRNA has a growth-promoting effect on T-cells [99] possibly by up-regulating the transcription of the E2F1 gene and its downstream targets. The importance of this function of hbz as a non-coding RNA is further reinforced by the recent finding of its localization mainly in the nucleus [62]. Interestingly, HTLV-1 does not encode a Vif ortholog, although a nucleocapsid portion of gag inhibits APOBEC3G restriction [100].

## 4. Expression Strategies of Other Human Complex Retroviruses

Spumaviruses, also referred to as foamy viruses (FVs), have been isolated from several primate species, cats, cattle and horses, and a few other mammals [101]. The first FV to be cloned and sequenced was isolated from human cells [102] and is referred to as the prototype FV; it probably represented a zoonotic infection of a chimpanzee virus. Although FVs are highly cytopathic in many cell types in tissue culture, leading to syncytium formation, vacuolization and cell death [102], they do not appear to be pathogenic *in vivo*.

Features that distinguish FVs from other complex retroviruses in terms of genetic organization, expression profile and replication strategy [103] include the use of a second plus-strand promoter in addition to the canonical LTR promoter, production of the Pol precursor protein from a spliced mRNA, and reliance on cellular factors rather than a Rev ortholog for nuclear export of incompletely spliced mRNAs [103].

The presence of 2 promoters permits a clear-cut temporal regulation of FV transcript expression. The internal promoter (IP) is located near the 3′end of the env gene and is responsible for expression of mRNAs coding for the regulatory/accessory proteins Tas (previously named Bel-1) and Bet [104]. Activity of both promoters is controlled by Tas, a strong transcriptional transactivator that binds to specific DNA sequences [105]. Early in infection, basal activity of the IP results in low levels of Tas protein which in turn binds to regulatory regions of the IP, resulting in increased promoter activity and enhanced Tas production. Following the rise in Tas concentration, the protein drives expression from the LTR promoter [106]. This results in production of the unspliced genomic/Gag transcript and singly spliced transcript coding for Env as well as a singly spliced transcript coding for Pol [107–109].

The production of Pol independently of Gag may lead to precocious reverse transcription; this might explain another intriguing feature of FV, *i.e.*, the production of virions which already contain proviral DNA genomes [110]. The role of Bet in FV replication is less well understood, although it has been shown to block APOBEC3 in some experimental systems [111,112].

Early studies of FVs indicated that they do not rely on a viral post-transcriptional regulatory protein such as Rev for expression [113]. However, a recent study of the prototype FV by Bodem *et al.* demonstrated its reliance on the CRM1 export pathway for nuclear export of the unspliced transcript. This process is mediated through binding of the cellular protein HuR to an as yet-unidentified element on FV RNA, with the presence of HuR on the RNA allowing engagement of the CRM1 pathway through recruitment of the HuR-CRM1 bridging proteins ANP32A and ANP32B [114].

Complex genetic structure and post-transcriptional regulation of viral gene expression are also characteristic of some human endogenous retroviruses (HERVs). A subset of HERVs termed HERV-K entered the germline of primates in relatively recent times (45 million year ago) [115] and are considered to be the most active HERVs, as they are capable of expression and generating virus-like particles [116]. The HERV-Ks are divided in type 1 and type 2 proviruses depending on the absence or presence of a 292 nt sequence at the boundary between the pol and env genes; the absence of this region in type 1 proviruses results in fusion of pol and env. Type 2 HERV-Ks generate 4 transcripts of 8.6 kb (full-length, encoding Gag-Pol), 3.3 kb (singly-spliced, encoding Env), 1.8 kb (doubly-spliced, encoding the Rec regulatory protein) and 1.5 kb (singly-spliced) [116]. Rec is a 14.7 kDa nucleolar protein that is functionally similar to HIV Rev and HTLV Rex. Rec function is mediated through its binding to a Rec Responsive Element (RcRE) located in the U3R segment of the 3′ LTR. Similar to Rev and Rex, Rec enhances stability and nuclear export of the unspliced and singly spliced mRNAs [117]. Rec is expressed in normal testicular cells and at much higher levels in germ cell testicular cancers. Consistent with this finding, Rec exhibits oncogenic properties in mice [118]. Type 1 HERV-K proviruses, which do not express Rec, express Np9, a 9 kDa protein [119] that shares 15 N-terminal amino acids with Rec while the C-terminal 59 residues are encoded in a different reading frame. It is noteworthy that most malignant tissues express Np9 while no Np9 transcripts are generated in normal human tissues. In the case of some tumors (e.g., breast cancer) expression of type 1 over type 2 HERV-K appears to be predominant [120].

## 5. Open Questions and Perspectives

Although considerable knowledge has been gained on the key features and regulatory mechanisms of retroviral gene expression, important questions remain to be addressed.

The biological significance of HIV-1’s complex and apparently redundant splicing pattern remains to be understood. Furthermore, the relative abundance and the time course of expression have not been measured at a single transcript level with quantitative splice-site-specific methods such as those used for studies of HTLV-1.

Although the cellular machinery involved in Rex and Rev function appears to be similar, possible peculiarities are suggested by the nonreciprocal complementation of Rex and Rev function as well as by the positioning of the *cis*-acting responsive elements; while the HTLV-1 RXRE is present in all the positive strand viral mRNAs regardless of their Rex-dependence, in the case of HIV, the RRE element is present on the Rev-responsive transcripts and absent from the Rev-independent mRNAs.

The mechanisms determining splice site selection in HTLV-1 and their possible connection with the differentiation and/or activation status of the host cell are largely unknown. The Rex-dependency of the alternatively spliced mRNAs encoding the accessory proteins (which do not contain any known *cis*-acting inhibitory elements) is not known at present. Furthermore, it is still unclear whether different patterns of viral gene expression/latency may lead to different clinical outcomes (no disease, ATLL or TSP/HAM). In addition it would be interesting to compare the levels and kinetics of mRNA expression of HTLV-1 to that of HTLV-2, a highly related deltaretrovirus with complex genetic organization and expression strategies. It would be most informative to carry out these studies using primary PBMCs from HTLV-2-infected patients, as the detection of a temporal switch in the expression of individual viral transcripts with splice site-specific Real Time RT-PCR has proven difficult to demonstrate in other systems [60,61].

Although both HTLV-1 and HTLV-2 immortalize T-cells in culture and establish persistent infections *in vivo*, the 2 viruses exhibit distinct pathogenic properties, as HTLV-2 has not been conclusively linked to lymphoproliferative or neurodegenerative diseases [121]. Differences in the pattern and time course of expression of the two viruses could thus provide molecular grounds to explain their different pathobiology.

The reliance of HIV on alternative splicing and its engagement of the Rev-CRM1 export pathway are already being exploited for the design of therapeutic agents that disrupt splicing or interfere with Rev function [17]. Given its similarities to HIV, similar strategies could be pursued for controlling HTLV-1 infection and pathogenesis.

## Figures and Tables

**Table 1.** Functional characteristics of human immunodeficiency virus-1 (HIV-1) accessory proteins.

**PROTEIN**	**LOCALIZATION**	**FUNCTION**
Nef	cytoplasm, nucleus, virion	enhances clathrin-mediated endocytosis and degradation of CD4; downregulates surface expression of MHC class I and T cell receptor (TCR)-CD3 complexes
Vpu	cytoplasm, nucleus	favors CD4 retention in the endoplasmic reticulum and proteasomal degradation; promotes release of nascent virions from infected cells by inhibiting tetherin
Vif	cytoplasm, virion	induces proteasomal degradation of the APOBEC3G restriction factor
Vpr	nucleus mitochondria, virion	exerts cytopathic effects through its ability to affect mitochondrial function; increases viral transcription and induces cell-cycle arrest in the G2 phase

**Table 2.** Functional characteristics of HTLV-1 accessory proteins.

**PROTEIN**	**LOCALIZATION**	**FUNCTION**
p30Tof	nucleolus, nucleus	inhibits nuclear export of the tax/rex mRNA; affects Tax-mediated transcription; affects the expression of cellular genes; interacts with Rex; interferes with TLR4 signaling
p21Rex	cytoplasm	represses Rex in some experimental systems
p13	mitochondrial inner membrane, nucleus	alters mitochondrial K^+^ permeability and increases mitochondrial ROS production; activates normal resting T-cells while promoting death of transformed cells; exerts antitumor effects *in vivo*; inhibits Tax function in the nucleus
p12	endoplasmic reticulum, Golgi apparatus	binds to the IL-2R β and γ chains; sequesters free MHC-I heavy chains; interacts with calreticulin and calnexin resulting in Ca^2+^ release from the ER and NFAT activation
p8	cell surface, immunological synapse	recruited to the immunological synapse; increases T-cell contacts through LFA1 and intercellular conduits
HBZ	nucleus	HBZ protein: inhibits Tax, Jun-B and c-Jun; stimulates Jun-D; HBZ RNA: growth-promoting effects in T-cells
